# Clinical Manifestation, Dermoscopy, and Scanning Electron Microscopy in Two Cases of Contagious Ecthyma (Orf Nodule)

**DOI:** 10.1155/2018/2094086

**Published:** 2018-10-29

**Authors:** Ana Laura Rosifini Alves Rezende, Fred Bernardes Filho, Natália Aparecida de Paula, Loan Towersey, Roderick Hay, Marco Andrey Cipriani Frade

**Affiliations:** ^1^Dermatology Division, Department of Internal Medicine, Ribeirão Preto Medical School, University of São Paulo, Ribeirão Preto, São Paulo, Brazil; ^2^AIDS Division, Carlos Tortelly Municipal Hospital, Ministry of Health, Niterói, Rio de Janeiro, Brazil; ^3^International Foundation for Dermatology, London, UK

## Abstract

Orf is a highly contagious skin disease commonly seen in goats and sheep that can be transmitted to people who have direct contact with infected animals. Here, we report the clinical manifestation, dermoscopy, and scanning electron microscopy in two women who developed skin lesions on their hands after handling goats with wounds in the udders. Human orf is usually self-limiting and no specific treatment is needed.

## 1. Introduction

Contagious ecthyma also called contagious pustular dermatitis or scabby mouth is a zoonotic disease, called orf in humans, which is caused by a double-stranded DNA virus, ORFV, and usually affects sheep and goats [1, 2]. Human infection occurs through inoculation of broken or abraded skin from infected animals or contaminated fomites [1, 3].

The clinical manifestation, dermoscopy, and scanning electron microscopy in two women, who developed skin lesions on their hands after handling goats with wounds on the udders, are presented herein.

## 2. Case Report

**Figure 1:**
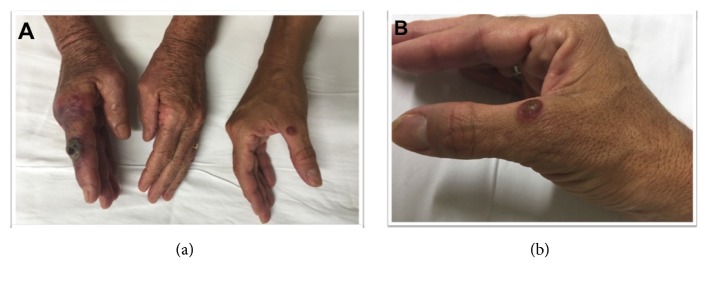
(a) Erythematous-violaceous papules and an ulcerated violaceous nodule on the patient's (left) and her daughter's (right) fingers; (b) a violaceous erythematous lesion with red outer ring, and erythematous border on the right thumb of patient's daughter.

A 63-year-old female came for consultation presenting with an erythematous violaceous plaque on the right index finger that had started 7 days previously. On examination a central necrotic area (Figure 1(a)) was observed. Fracture and acute vascular occlusion were excluded. Laboratory tests were unremarkable. A consultation with the dermatology department was then requested.

**Figure 2:**
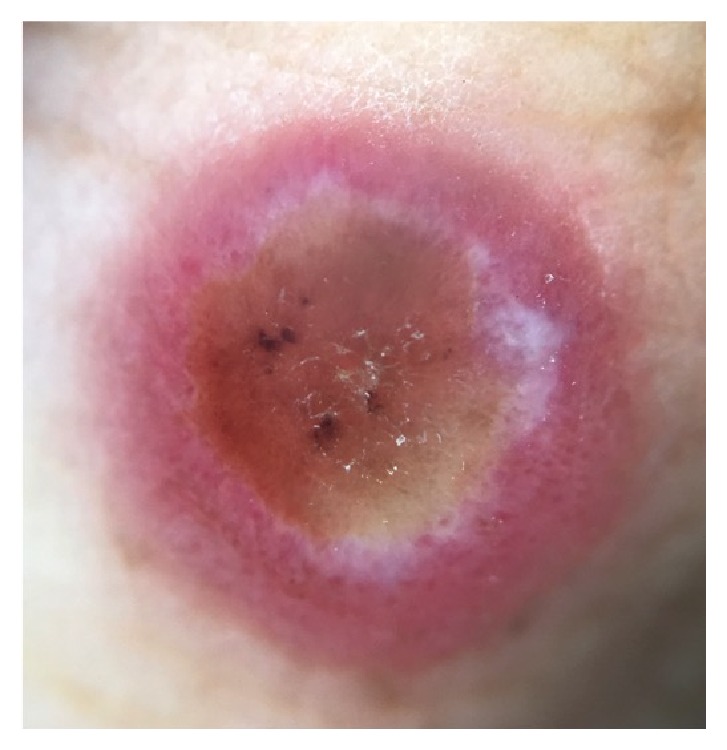
Dermoscopy showed an erythematous area, central ulceration, yellow crust, brown dots, a white structureless zone partially surrounding it, and dotted vessels; original magnification x10 (DermLite II Pro 3Gen, San Juan Capistrano, CA, USA).

The patient raised goats on her farm. The animals had some udder lesions, so she needed to daily bottle-feed milk to the kids. She did not wear gloves while performing this task. During the evaluation of the patient, it was observed that the patient's daughter presented with a similar skin lesion on the left thumb, and she reported that she also helped to feed the little goats. Dermatology exam showed an indurated nodule with central umbilication covered by crust and surrounded by a reddish halo (Figure 1(b)). Dermoscopy of the finger nodule showed an erythematous area, central ulceration, yellow crust, brown dots, a white structureless area partially surrounding the lesion, and dotted vessels (Figure 2). The diagnosis of orf was suspected.

**Figure 3:**
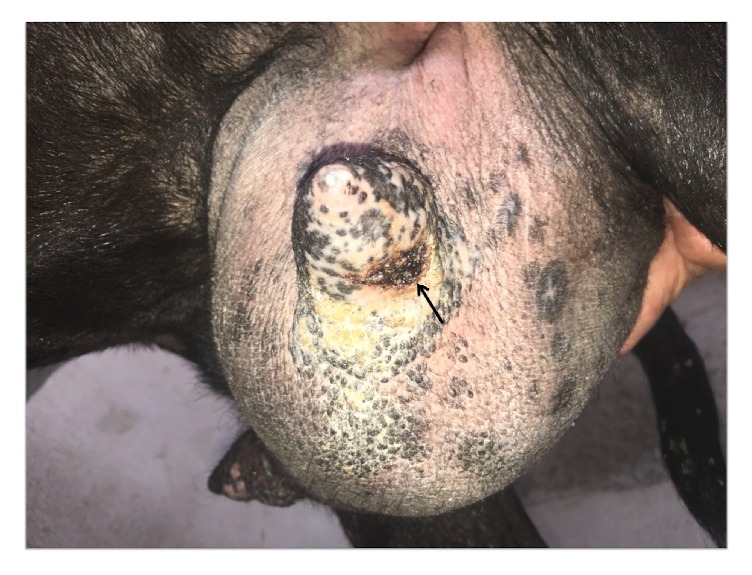
(a) Udder lesions (arrow) on the patient's goat.

**Figure 4:**
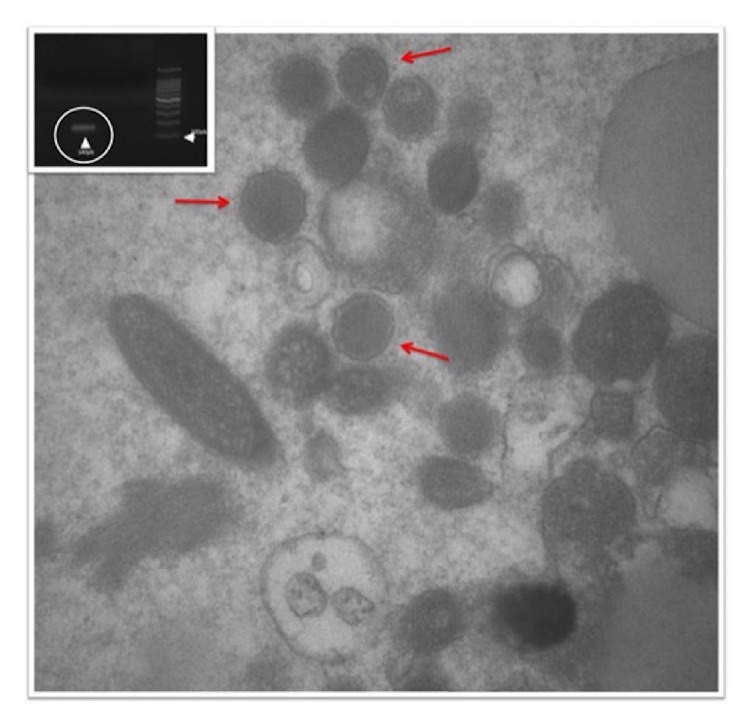
Electron microscopy showing numerous viral particles (DNA core surrounded by double layered capsid) (red arrow). Insert: polymerase chain reaction (PCR) orf virus result. 2% agarose gel, with PCR product with ORF1/ORF2 primers, specific for orf virus; 140pb positive fragment (white circle); DNA ladder (100pb).

**Figure 5:**
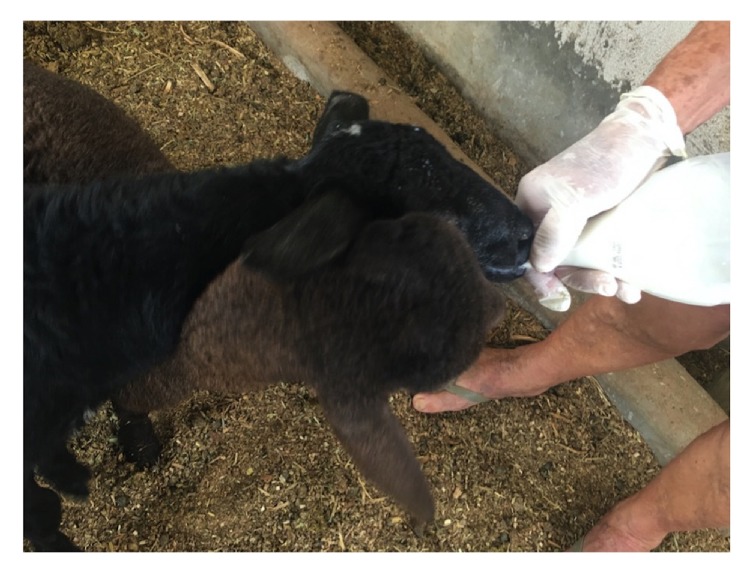
Patient feeding the kids using gloves.

Upon domiciliary visit to the patient's farm, goats with udder lesions (Figure 3(a)) were found. The electron microscope has been used for the diagnosis of orf. In this case, it showed ovoid particles with a crisscross appearance due to viral particles (Figure 4); polymerase chain reaction was positive for the specific virus (ORFV) (Figure 4). The patient was advised to feed the kids using gloves (Figure 5) and to commence local wound care for the lesions, because the disease was showing spontaneous regression.

## 3. Discussion

Orf is characterized by one or multiple nodules on the hands and fingers, but also on the feet, legs, neck, and face [1–3]. The disease passes through different phases. The first phase occurs after a brief incubation period of 3 to 5 days and presents with a small papule. Then, the lesions enlarge and progress to nodules that ulcerate and form crusts. The disease usually does not require specific treatment, because the lesions show spontaneous regression within 4–8 weeks [1–3]. Besides dermatologic features, the patient may experience some systemic symptoms and signs, such as fever and, less commonly, lymphangitis, lymphadenitis, and ocular damage [2, 3]. Orf may also trigger erythema multiforme [4].

The diagnosis is based on the history, physical examination, and some complementary investigations, such as dermoscopy, histopathology, PCR detection, and electron microscopy [1, 5–7].

Occupational skin diseases are particularly important in dermatology, because they can lead to high morbidity in workers and can also reduce their productivity. Moreover, they also represent a public health risk.

The case of orf nodules in relatives shows the importance of disseminating knowledge about this skin disease, because the patient was sent to the emergency room due to suspected necrosis of the finger, but actually she had a benign and self-resolving disease.

Although no person-to-person spread occurs, people in the same environment may be contaminated by the same source (infected animal). Differential diagnoses include, depending on the phase of the disease, anthrax, atypical mycobacteriosis, cowpox, pseudocowpox (Milker's nodule), pyoderma, herpetic whitlow, tularemia, keratoacanthoma, fish-tank granuloma, and sporotrichosis [1, 2, 8]. Dermoscopy is a very helpful tool to diagnose orf and Milker's nodule, but it cannot differentiate between them, so further diagnostic tools are required. We used both electron microscopy and PCR detection to confirm the orf virus diagnosis.

We emphasize the importance of using more specific techniques to confirm the diagnosis, especially when dealing with an occupational skin disease which has repercussions on public system.
